# Composition and Quality of Honey Bee Feed: The Methodology and Monitoring of Candy Boards

**DOI:** 10.3390/ani14192836

**Published:** 2024-10-01

**Authors:** Soraia I. Falcão, Michel Bocquet, Robert Chlebo, João C. M. Barreira, Alessandra Giacomelli, Maja Ivana Smodiš Škerl, Giancarlo Quaglia

**Affiliations:** 1 Centro de Investigação de Montanha (CIMO), Instituto Politécnico de Bragança, Campus de Santa Apolónia, 5300-253 Bragança, Portugal; jbarreira@ipb.pt; 2 Laboratório Associado para a Sustentabilidade e Tecnologia em Regiões de Montanha (SusTEC), Instituto Politécnico de Bragança, Campus de Santa Apolónia, 5300-253 Bragança, Portugal; 3 Apimedia, 82 Route de Proméry—Pringy, 74370 Annecy, France; apimedia@aol.com; 4 Faculty of Agrobiology and Food Resources, Slovak University of Agriculture, Tr. A. Hlinku 2, 949 76 Nitra, Slovakia; robert.chlebo@uniag.sk; 5 UNAAPI—Italian National Union Beekeeper Association, Via Paolo Boselli 2, 50136 Florence, Italy; alessandra.giacomelli@unaapi.it; 6 Agricultural Institute of Slovenia, Hacquetova Ulica 17, SI-1000 Ljubljana, Slovenia; maja.smodis.skerl@kis.si; 7 Lifeanalytics—FLORAMO, Via Pezza Alta 22, 31046 Oderzo, Italy; giancarlo.quaglia@lifeanalytics.it

**Keywords:** honey bee nutrition, candy boards, supplements, bee health

## Abstract

**Simple Summary:**

The nutritional status of honey bee colonies is a crucial point for honey bee colony health, as inadequate nectar and pollen flow can lead to colony development issues, increased pathogen proliferation, and reduced colony activity and strength. Beekeepers are thus encouraged to use food supplements or substitutes to correct nutritional imbalances. With a growing market driven by minimal regulation and beekeeper demand, the market offers numerous bee food supplements with varying and sometimes undefined compositions, claiming benefits such as brood stimulation, energy supplementation, and disease prevention. Through the analysis of a short set of physico-chemical characteristics, it was possible to find differences between the values found and the ones displayed on the label, demonstrating the need for regulation of the quality of these products.

**Abstract:**

The nutritional status of a honey bee colony is recognized as a key factor in ensuring a healthy hive. A deficient flow of nectar and pollen in the honey bee colony immediately affects its development, making room for pathogen proliferation and, consequently, for a reduction in the activities and strength of the colony. It is, therefore, urgent for the beekeepers to use more food supplements and/or substitutes in apiary management, allowing them to address colony nutritional imbalances according to the beekeeper’s desired results. In this context, the commercial market for beekeeping products is growing rapidly due to low regulation of animal food products and the beekeeper’s willingness to guarantee healthy colonies. There are numerous products (bee food additives) currently available on the worldwide market, with a highly variable and sometimes even undefined composition, claiming a set of actions at the level of brood stimulation, energy supplementation, queen rearing support, reduction of Varroa reproduction levels, improvement of the intestinal microflora of bees, Nosema prevention, and improvement of the health of honey bee colonies infested by American foulbrood, among others. To address this issue, the members of the COLOSS (Honey Bee Research Association) NUTRITION Task Force are proposing, for the first time, action on honey bee feed control and monitoring. In our common study, we focused on candy board composition and quality parameters. For that, a selected number of commercial candy boards usually found in Europe were analyzed in terms of water and ash content, pH, acidity, 5-hydroxymethylfurfural, sugars, C3-C4 sugar origin, and texture. Results revealed differences between the values found and the ones displayed on the label, demonstrating the need for regulation of the quality of these products.

## 1. Introduction

Nutritional stress due to habitat transformation and loss is thought to be among the major factors contributing to current declines in bee populations. Understanding bee nutrition is critical to overcome population decline [1]. The bees feed on a number of substances from nature that ensure their survival, namely nectar, pollen, and water. Both adult bees and larvae are highly dependent on the colony’s food reserves, where adult bees may change the harvesting management and rearing strategies according to specific carbohydrate and protein needs. A balanced diet is the basis for the colony’s growth and development [1,2]. Nectar is the main source of carbohydrates used for energy production, which can be converted and stored as body fat. The presence of fresh nectar in a colony also serves as a stimulus to the expansion of the brood area and, consequently, to colony growth. Nectar collected in the flowers is mainly composed of sucrose, which, after the addition of enzymes, is converted into fructose and glucose. These sugars are easily digested by bees, as well as other saccharides such as maltose, trehalose, or melezitose; however, the presence of galactose, mannose, lactose, raffinose, and other polysaccharides can cause toxicity, particularly if administered in high doses, leading to mortality of the bees [3]. The consumption of carbohydrates occurs at all stages of bee development: during the larval stage, the consumption of these substances increases from 18% in the initial phase to 45% in the last two days of development [1]. In adulthood, the diet is almost exclusively based on these substances, requiring about 4 mg of available sugar to survive. The absence of an adequate flow of nectar has a direct impact on the colony, increasing its aggressiveness, decreasing hygienic behavior, reducing the rearing area, reducing the bees’ lifetimes, and ultimately causing the mortality of the colony by starving [3]. The main causes of the lack of carbohydrates are associated with long periods without the possibility of foraging (rain and low temperatures) or an imbalance in the amount of population related to nectar intake (prolonged rains after flowering or after honey removal). Under these conditions, it is up to the beekeeper to act on the colony using artificial feeding in the colonies. Additionally, there may also be a need to supply carbohydrates during the process of producing new colonies or when it is desired to artificially stimulate the amount of rearing to prepare the colony for the next flowering.

Artificial carbohydrate supplementation is usually carried out by feeding bees honey pastes and syrups, sucrose, invert sugars, and high fructose corn syrups (HFCS) [4,5]. This supplementation can present, however, some risks. One of the possible risks may be honey adulteration: in pure honey, the sugars are derived almost from nectar provided by C3 plants. However, honey bee nutrition can be realized either with C3 or C4 plant sugars, and their presence can be detected in honey by the official stable carbon isotope ratio AOAC method No. 998.12 [6]. The stable carbon ratio value of the whole honey is compared to the stable carbon isotope ratio value for protein isolated from honey. The difference between these values is a measure of the C4 sugar content of honey. The maximum difference tolerated is −1%, corresponding to a C4 sugar content of 7%; otherwise, the honey is considered adulterated [6]. Cabanero [7] and Elflein and Raezke [8] improved the detection method of addicted C3 invert sugars by the coupling of an isotope ratio mass spectrometer both to an elemental analyzer and to a liquid chromatograph (EA/LC-IRMS). If, on the one hand, the addition of honey syrups appears as the most obvious supplement, it is necessary to consider the origin of the honey since it can act as a vector of transmission of certain bee diseases. On the other hand, the use of honey from the extraction process remains or after long periods of storage may present an increased risk due to the formation of a degradation product, hydroxymethylfurfural (5-HMF), which, above 30 ppm, is toxic to the bees [9,10,11,12,13,14,15]. This parameter is also one of the risk factors for the use of HFCS in artificial feeding, not only because of its storage but also because of the production process. Another risk factor is the introduction of enzymes that result from the syrup production process, as well as the presence of high amounts of polysaccharides, which are toxic to bees [13]. Researchers use field-collected bee samples to obtain information on the quality of nutritional resources available to bee colonies [16,17]. Measures of worker bee head and thorax weight are indicators of nutrient assimilation into brood food-producing head glands and flight muscles, respectively [18]. Molecular biomarkers such as mRNA expression of the storage protein Vitellogenin (Vg) have been used to assess bee nutritional status because Vg levels are linked to diet quality [19]. Nutrition also induces changes in the honey bee gut microbiota, with consequences on host immune function and pathogen susceptibility [20]. Microbiota abundance is, therefore, a potential biomarker of honey bee disease and nutritional status that warrants further investigation [21]. Scientists all over the world have formulated different artificial food recipes for bees on the basis of the nutrient composition of honey and pollen, acceptability, palatability, digestibility, and affordability of ingredients. This may help to maintain all colony parameters enough to derive maximum advantage of the forthcoming floral-rich season [22]. There are several studies summarizing and evaluating the efficacy and forms of sugar syrups [10,23,24,25,26] and dry sugar [27], but there is still a lack of information on the production procedure, composition, safety, and efficacy of commercially available carbohydrate candy boards [15,28,29,30].

In the frame of the COLOSS (Honey Bee Research Association) NUTRITION Task Force [31], we propose action on honey bee feed control and monitoring, setting four main objectives: elaborate methodologies to study bee aliments (protocols, good laboratory practices) and implicate different stakeholders to clarify the type of analyses depending on their needs (e.g., organic or legal framework); create and coordinate a network of laboratories able to use the proposed methodologies (ring tests, evolution of the methods with new technologies); apply the methodologies to a large set of bee ailments at a worldwide scale; and elaborate guidelines to support and assist food companies and regulators to effectively control the quality and safety of supplements and substitutes for honey bees.

The main objective of this study was to analyze selected candy boards of different origins found in the European market. Candy boards are a source of food for bees in which a form of hardened sugar mixture is usually placed on top of the frames in the spring period to enhance bee colony growth or as an emergency winter feeding [5]. For that, a set of parameters (water content, ash, pH, hydroxymethyl furfural (5-HMF), sugar content, C3-C4 sugar origin, and texture) were determined in the different sugar candies. The common methodologies were performed in different laboratories to validate and compare the results obtained. Globally, this action will allow more information to the stakeholders via monitoring and setting the basis for the regulation of products to reach the minimum standards for quality, effectiveness, and economy of honey bee feed, finally guaranteeing the quality of the bee products.

## 2. Materials and Methods

### 2.1. Participants

Water content, ash, acidity, pH, hydroxymethyl furfural (5-HMF), and sugar analyses were performed in a collaborative study accomplished by 3 laboratories established in 3 countries (Portugal, Slovakia, and Slovenia), while C3-C4 sugar origin was performed in a laboratory established in Italy. The different analyses were performed in triplicate. All the research laboratories had different levels of experience in bee products and/or food analysis.

### 2.2. Standards and Reagents

All analytical grade reagents and chemical standards were obtained from Sigma Chemical Co. (St. Louis, MO, USA). HPLC-grade methanol, ethanol, and acetonitrile were purchased from Fisher Scientific (Leicester, UK). Water was treated in a Milli-Q water purification system (TGI pure system, Houston, TX, USA).

### 2.3. Samples

For this study, 8 commercial honey bee candy boards frequently found in Europe were analyzed (Table 1). After the candy board purchase, each sample was divided into equal parts and sent to the laboratories participating in the experimental analysis. The candy boards were kept confidential to prevent the disclosure of the obtained information. All the samples were within the warranty period and were stored in accordance with the instructions.

### 2.4. Water Content

For the determination of the water content, the AOAC 925.45 was followed [32].

### 2.5. Ash Content

The ash content of the samples was estimated through the AOAC 900.02 [33].

### 2.6. pH Determination and Free Acidity

These parameters were determined, following the IHC method [34], by titration to pH 8.3.

### 2.7. 5-Hydroxymethylfurfural (5-5-HMF)

The determination of 5-5-HMF was performed by liquid chromatography (HPLC) with UV detection in accordance with the procedures described by the International Honey Commission [34]. A total of 10 g of sample was weighed into a 50 mL beaker. The sample was dissolved in water and transferred quantitatively to a volumetric flask of 50 mL. After, it was filtered through a 0.45 µm membrane filter to a vial for chromatography. The mobile phase was water/methanol (90:10), with a flow rate of 1 mL/min and an injection volume of 20 µL. The detection was performed at 285 nm. For the standard calibration, 5-5-HMF standards with concentrations of 1, 2, 5, and 10 mg/L aqueous solution were prepared. For the determination of the standard 5-HMF content, the absorbance A of the prepared standard solution was determined using a UV spectrophotometer at 285 nm in 1 cm quartz cells with water in the blank cell. The concentration of the standard solutions was calculated using the molar absorptivity, ε = 16,830, or absorptivity, a^1%^_1cm_ = 133.57.

(1) $$\mathrm{Concentration}\;\mathrm{in}\;\mathrm{mg}/\mathrm{mL}=\frac{A}{1\times 133.57}\times 1000$$

The 5-HMF content of the sample was calculated by comparing the sample’s peak area with those of the standard solutions, taking into account the dilution. The results were expressed in mg/kg.

### 2.8. Determination of Sugars by HPLC-RI

The sugar content was measured using liquid chromatography coupled to a refraction index detector (HPLC-RI, Berlin, Germany) in accordance with the procedures described by the International Honey Commission [34]. The samples were prepared by diluting 5 g of candy patty with 40 mL of distilled water. Next, 25 mL of methanol was pipetted into a 100 mL volumetric flask, to which the honey solution was then added. Lastly, water was added to m ake up the final volume. The solutions were filtered through a membrane filter and collected in sample vials. As a mobile phase, a mixture of acetonitrile/water 80:20 (*v*/*v*) was used, with a flow rate of 1.3 mL/min and an injection volume of 10 µL. An analytical stainless steel column, e.g., 4.6 mm in diameter and 250 mm in length, containing amine-modified silica gel with 5–7 µm particle size, was used. The identification of the sugars was obtained by comparing the retention times of the sample peaks with those of standards, and quantification was performed using standard samples of known composition and concentration.

### 2.9. C3-C4 Sugar Origin

The stable carbon isotope ratio δ13C values for C4 plant origin sugar evaluation were measured according to AOAC 998.12 Method with a Flash 2000 EA coupled to a Delta V Advantage (Thermo Fisher, Bremen, Germany) via a ConFlo IV interface. Briefly, the EA was operated with a 100 mL min^−1^ helium flux and temperatures of 950 °C in the oxidation tube and 850 °C in the reduction tube. The outlet was equipped with a column that physically retained CO_2_ at 70 °C; CO_2_ was released by increasing the temperature to 210 °C. The overall experiment duration was 600 s. The δ13C values (‰) were calibrated to Vienna Pee Dee Belemnite with three pulses of CO_2_ reference gas and then calibrated against the international standard. Calibrations were performed at the beginning of the elution run. Samples were weighed in tin capsules. The evaluation of δ13C values of saccharides present in candy was performed by combining chromatographic separation of candy saccharides by liquid chromatography and IRMS (LC-IRMS), according to Elflein and Raezke [8], with a Thermo Surveyor HPLC coupled to Delta V Advantage (Thermo Fisher, Bremen, Germany) via an LC-ISOLink interface. Candy was diluted with water, filtered, and injected into a liquid chromatography system for separation into mono-, di-, tri-, and oligosaccharides using only water as mobile phase and Phenomenex Rezex RCM-Monosaccharide Ca^2+^ (8%), 300 × 7.8 mm column thermostated at 50 °C. The column effluent is fed into an interface LCISOLink where organic compounds are oxidized to CO_2_ by wet digestion with a solution of Natrium Persulfate 0.5 M/Phosphoric Acid 0.5 M. CO_2_ isotopologues with *m*/*z* 44, 45, and 46 are separated in the spectrometer and detected using Faraday cups. Compound specific δ13C (‰) values are then calculated according to Formula (2):

(2) δref C13/C12=Rsample  C13/C12Rreference C13/C12−1

See below, Table 2, with the δ^13^C isotopic range of some C4 and C3 plant origin.

### 2.10. Texture

The texture profile was conducted using a Stable Micro Systems (Vienna Court, Godalming, UK) TA.XT Plus texture analyzer with a 30 Kg load cell. The probe used was the P/2 (2 mm cylinder) probe, which was used to perform a penetration test, allowing the determination of the hardness of the samples. The pre- and post-test speeds were set at 2mm/s, and the target mode was set to distance that started at 10 g of force. The results were combined and processed through a macro and analyzed through the Exponent program.

### 2.11. Statistical Analysis

For each tested beefed candy bar, three independent samples were analyzed, and each sample was analyzed three times. Data were expressed as mean ± standard deviation. The statistical tests were applied considering a value of α = 0.05 (95% confidence), using the IBM SPSS Statistics for Windows software, version 26.0. (IBM Corp., Armonk, NY, USA).

An analysis of variance (ANOVA) with type III sum of squares was performed using the GLM procedure (Generalized Linear Model). All dependent variables were analyzed using 2-way ANOVA, considering candy boards (CBs) and laboratory (Lab) as variability factors. Once there was a significant interaction between the two factors in all cases, the results were compared using the graphs of the estimated marginal means. Compliance with ANOVA requirements, specifically the normal distribution of results and the homogeneity of variances, was verified using the Shapiro–Wilk and Levene’s test, respectively.

A linear discriminant analysis (LDA) was also carried out to globally characterize each bee feed candy board. The variables were selected sequentially (stepwise), considering the Wilks’ ʎ test with the usual F probabilities (3.84 to enter and 2.71 to remove). This procedure is based on the simultaneous verification of the significance of all previously selected variables before the inclusion of a new one. The main objective was to estimate the relationship between the dependent categorical variables (CB) and the quantitative independent variables (results obtained for the quality parameters). To assess the performance and adequacy of the discriminant model, an internal cross-validation procedure was applied.

## 3. Results

Quality indicators are shown in Table 3. Values presented for the levels of each factor were obtained by the mean values of all levels of the other factor, thereby justifying the high standard deviation values of each laboratory, which is simply a result of the heterogeneity of the analyzed CB. In turn, the low standard deviation values obtained for each CB are a good indicator of the precision among laboratories, validating the obtained outcomes. As it might be observed, the interaction among factors was significant in all cases, not allowing the values to be classified according to the Tukey (or Tamhane’s T2) test. Nonetheless, it is obvious that CB S-6 presented significantly (*p*-value < 0.001) higher acidity, followed by S-7 and S-4. CB S-6 stood out as well in sucrose content, as this was the only sample in which this individual sugar was absent, while all the remaining samples presented sucrose concentrations around 75 g/100 g of CB. In turn, S-6 showed the highest quantities of fructose (32 ± 1 g/100 g of CB) and glucose (40 ± 1 g/100 g of CB), while both sugars lay below 10 g/100 g of CB in all other studied samples. Except for candy S-2, 5-5-HMF values were correlated with acidity (and, obviously, pH) values, as evidenced by the maximum obtained for S-6 (58 ± 5 mg/kg CB) and the lowest values observed in S-1 (3.0 ± 0.5 mg/kg CB), S-3 (1.3 ± 0.4 mg/kg CB), S-5 (3.2 ± 0.5 mg/kg CB), S-8 (1 ± 1 mg/kg CB), and S-9 (2.2 ± 0.5 mg/kg CB). Lastly, S-6 also showed a significant difference in its water content (14 ± 1 g/100 g of CB), which was approximately three-fold higher than in all other CB. For the 5-HMF, the values found indicate improper storage of the candy boards at room temperature, which can lead to unacceptable levels within a few months.

**Figure 1:**
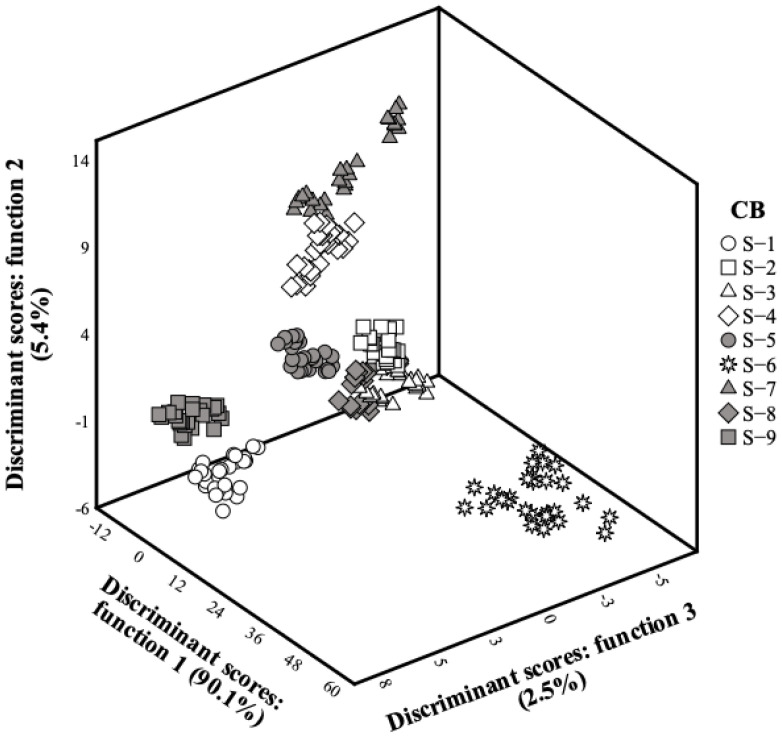
Three-dimensional distribution of CB markers according to the discriminant function coefficients defined from the assayed quality parameters.

After studying each CB separately, a linear discriminant analysis was performed to verify the differences among different CBs from a global perspective. As it might be concluded from Figure 1, function 1 mostly separated the markers corresponding to S-6, and the highest correlation coefficient (0.805) was obtained for fructose. Function 2, on the other hand, separated mostly S-4 and CB S-7, being particularly correlated (0.828) with acidity values, which, apart from S-6, had maximal values in these CBs. Function 3 was effective in separating S-1 and S-9, mostly due to its correlation with fructose (0.760), which, once again, except for CB S-6, showed the highest values in these CBs.

Considering the higher percentage of variability explained by function 1 (90.1%), it became quite evident that S-6 was clearly different from all the remaining samples. Nonetheless, other conclusions could be obtained as well, for instance, in what concerns choosing the CB with the highest fructose (S-3, S-6, and S-9) or avoiding those with the highest acidity (S-4, S-6, and S-7).

On the basis of isotopic EA/LC-IRMS analysis results, Table 4, sample S-6 is the only candy board sample of pure C4 plant sugar origin (probably from maize or cane sugar): the value of δ13C is in the range of C4 plant sugar origin (see Table 2 with the example of the δ^13^C isotopic range of C4 and C3 plant origin); other samples (S-1, S-3, S-4, S-9) are C3-based sugar plant origin with a part of C4 plant sugar origin; the remaining samples (S-2, S-5, S-7) are pure C3 based sugar plant origin.

The results of the texture profile analysis are given in Table 5. The hardness was the parameter evaluated, which is normally related to moisture and fat content. The candy boards with the highest values were S-3 and S-4, with 528.8 ± 7.1 and 474.4 ± 28.7 hardness/g, respectively. Candy board S-6 showed the lowest value of hardness, with a value of 35.6 ± 6.3. The softer nature of this bee feed can be related to the high percentage of water present in this sample when compared to the others (Table 3).

## 4. Discussion

Concerns regarding the shortened lifespan of bees after consuming candies containing 5-HMF are supported by Skerl and Gregorc [15] and Zirbes et al. [35]. Studies on the production and composition of carbohydrates in candy boards are limited, with little comparison between various producers [28,29,30]. The main factors involved in the formation of 5-HMF are the material origin, temperature, pH value, and time. 5-HMF in bee food increases as the temperature used in its production increases, storage conditions, as well as the acids used in the inversion of sugars. The temperature during storage should be lower than 25 °C, and at room temperatures above 40 °C, 5-HMF levels should rise significantly. The 5-HMF level can rise from the normal low initial values of 25 mg/kg to over 350 mg/kg of carbohydrate feed. Due to this characteristic, the variation in composition should be minimized as much as possible. Abnormally high losses of bee colonies fed with syrups were recorded in several European countries in the recent past, where the inversion of sugars was supported by acids (or lemon juice), which subsequently potentiated the increase in 5-HMF [35]. At concentrations of 5-HMF above 350 mg/kg in bee feeding, the death of bee colonies was close to 100%. The homemade method of producing invert syrups in an acidic environment is highly risky for bees, even in industrially produced bee feed, the amount of 5-HMF must be monitored (the homemade method of producing inverted syrups in an acidic environment is highly risky for bees even in industrially produced bee feed, the amount of 5-HMF must be monitored) [10,35].

The 5-HMF present in bee feed is an undesirable substance. Immediately after carbohydrate feed production, 5-HMF levels are usually between 15 and 25 mg/kg. A legally binding maximum limit has not yet been established when determining the levels of 5-HMF in feed through analytical tests; therefore, it is necessary to examine the requirements for feed safety according to Article 15 of Regulation (EC) No. 178/2002. When feeding bees, the 5-HMF content (at 72% dry matter content) should not exceed 60 mg/kg of feed syrup. Increased 5-HMF in some of our samples is a result of storage at room temperature, although all the samples used in this experiment were within the expiration date. Recommendations for proper preservation and storage of candy boards were missing in most cases. Further, 5-HMF levels may also increase in the hive after the honey bees are fed and ultimately be present in the winter storages. The quantity can be affected by the duration of feed present in the hive and the conditions of how the honey bees start the winter (outdoor temperatures above 20 °C increase the level of 5-HMF) [35].

Regarding Lab effect, the only single parameter that showed an apparent difference among the three laboratories was water content, which tended to be quantified in lower concentrations in the Portuguese laboratory.

Supplemental feeding poses significant financial and labor burdens for large-scale beekeepers [34]. The market for beekeeping products has rapidly expanded to meet these demands, driven by minimal regulation of animal food products and beekeepers’ commitment to maintaining healthy colonies. Numerous diet formulations have emerged worldwide, combining various ingredients and claiming benefits such as brood stimulation, energy supplementation, support for queen rearing, Varroa mite control, enhancement of bee intestinal microflora, Vairimorpha (Nosema) prevention, and improvement of hive health against American foulbrood, among others [22]. However, a universally accepted standard balanced diet for commercial beekeeping remains elusive [22,31].

Globally, when comparing the obtained results with the information displayed on the label, no agreement was found, especially in terms of the exact amount of the different parameters. Information regarding product composition, the botanical source of sugars, shelf life, and storage conditions of candy boards was scarce. This point is important because honey bees are food-producing farm animals and are subject to the relevant European feed safety legislation. Honey bee feed that is potentially dangerous must not be placed on the market. Feed syrup obtained from sucrose is feed raw material in the sense of Regulation (EC) no. 767/2009 mentioned in point 4.1.12 of part C of the Annex to Regulation (EU) no. 68/2013 as “sugar syrup obtained by processing sugar and/or molasses”. In contrast, feed syrup obtained from hydrolyzate of starch (corn or wheat) and candy boards with the addition of pollen are compound feed. Manufacturers of carbohydrate feeds should follow the recommendations: ensure optimal representation of individual sugars in the syrup; ensure that the ash content does not exceed 0.5 g/kg of dry matter; avoid acidification in the production; use a process with a precise control of temperature in order to avoid high 5-HMF content; indicate the transport and storage conditions on the package (dry, dark rooms with a temperature below 25 °C); and follow a HACCP protocol, with control of the main drivers of candy quality for each batch produced (e.g., pH, humidity, 5-HMF content).

For the beekeepers using carbohydrate feeding, it is important to be attentive to the following recommendations: avoid purchasing sweet solutions or candies that are not specifically intended for feeding bees, e.g., made from molasses, fruit juices, brown sugar, etc., because they all contain impurities that can cause bee diarrhea. Some foreign additives and most types of sugar are toxic to bees or contaminate honey; give preference to syrups and candies made from white refined sugar over starch hydrolysates [31]. During the production of syrups and candies from starch, a certain percentage of dextrins are always produced [31] (give attention to the compositional analysis in the label concerning the composition of sugars, 5-HMF level), and for the proportion of non-digestible sugar components in the feed, the level of 5-HMF in the feed should be as low as possible, not exceeding 60 mg/kg of syrup [35]. It is also important to avoid long storage times, avoid mixing the fresh syrup with old bee food that you have stored for a long time, and eliminate the risk of residual feeding in honey, especially if enzymes that are markers of honey adulteration were used for their production [34,35]. Some protein additives to feed, such as brewer’s yeast or milk powder, as well as dubious vitamin supplements, may contain substances in honey that are interpreted as markers of honey adulteration with external sugars [35]. Additionally, some of these substances may be allergens [36,37].

The isotopic analysis performed was relevant to consider the effect of bee feeding on the authenticity of honey produced after the administration of the candy board. In fact, if the bee feeding is not properly conducted (e.g., overfeeding or administration of candy board during nectar crops), it can reflect negatively on the honey authenticity, which shall consequently not be in accordance with the Codex Alimentarius on honey and the European Council Directive 2001/110/EC of 20 December 2001 relating to honey. Currently, on the basis of the scientific literature on isotopic δ13C value range for pure honey, it shall respect the following compliance value on isotopic analysis results [6]: n value of C4 sugar < 7%: the honey sample was not adulterated with C4 exogenous sugars, the value of C4 sugar ≥ 7%: the honey sample was adulterated with C4 exogenous sugars. Bee feeding must follow good management practices, avoiding the overlap with honey production season. N Interpretation of EA-LC-IRMS individual δ13C isotopic results: difference in Δ δ13C (F-G, fructose–glucose): not more than ± 1.00‰; difference in Δ δ13C (Max of each individual δ13C value determination by AOAC 998.12 and LC-IRMS): not more than ± 2.10‰. More data are required to establish regulations for carbohydrate feeding; define minimum standards for quality, effectiveness, and affordability of honey bee feed; and ultimately ensure the quality of bee products. In this study, we have focused on pure sugar candy boards, but there is a growing market with fortified candy boards, with extremely little information on bee colony performance and health.

## 5. Conclusions

The nutritional status of a colony is recognized as a key factor in ensuring a healthy hive. A deficient flow of nectar and pollen in the hive immediately affects its development, making room for the proliferation of pathogens and, consequently, for a reduction in activities and the number of bees in the colony. It is, therefore, natural for beekeepers to increase the demand for food supplements as adjuvants in the hive’s management, allowing them to address nutritional imbalances according to the beekeeper’s desired results. Supplemental feeding is a management strategy with significant financial and labor costs for large-scale beekeepers. The market for beekeeping products has shown an enormous capacity to respond to these needs, with a growing and aggressive escalation in the number of products sold because of the low regulation of animal food products and the beekeeper’s willingness to guarantee healthy colonies. Many diet formulations have been developed by combining different ingredients and examined by various workers all over the world for commercial beekeeping. These products have highly variable and sometimes even undefined compositions. However, a standard balanced diet for commercial beekeeping that is accepted worldwide is still awaited.

Focusing on one of the simplest bee feeds, the sugar candy board commonly found in European markets, through the analysis of a short set of physico-chemical characteristics, it was possible to discriminate different levels of quality among the commercially sold products, with the majority of products presenting differences with the information given on the label. Candy board S-6 exhibited the highest acidity (*p* < 0.001) and differed from others by lacking sucrose, while all other samples had ~75 g/100 g. The highest fructose (32 ± 1 g/100 g) and glucose (40 ± 1 g/100 g) content was shown by S-6, unlike other samples, which were below 10 g/100 g. Also, this sample presented the highest 5-HMF (58 ± 5 mg/kg) and water content (14 ± 1 g/100 g). Isotopic analysis indicated S-6 was derived from pure C4 plant sugar, while others were mixed with C3 and C4. Texture analysis revealed that S-6 had the lowest hardness (35.6 ± 6.3), correlating with its high moisture content.

We had concordant results among the different laboratories involved in this study, and for specialized parameters, the complementarity of the different teams permitted to enrich the results.

In the frame of the Coloss Nutri task force, we proved the possibility of developing an international network of laboratories to collaborate in order to help different stakeholders, like manufacturers, policymakers, and beekeepers, to understand the stakes of bee feed quality and fill the gaps of knowledge in order to help them to take adequate decisions and practices.

## Figures and Tables

**Table 1:** List of candy boards analyzed.

Sample Code	Label Information
S-1	Glucose/fructose syrup Sugars (97%), fiber (0.1%), ash (0.1%), sodium (0.02%)
S-2	Sucrose in dry substance (max. 83.0%), dextrose (app. 5.5%), fructose (app. 3.0), maltose (app. 2.5%), higher saccharides (app. 8.0%)
S-3	Inverted liquid sugar glucose syrup sucrose, fructose, glucose
S-4	Saccharose (75%), glucose syrup (16%), fructose (9%)
S-5	Water content (11%), pH (6); sugars in dry substance: sucrose (86%), fructose (3%), glucose (2%), other sugars (9%)
S-6	Glucose, fructose, other sugars
S-7	Sucrose (77.28%), fructose (6.08%), glucose (6.01%) Total sugars (90.52%), water content (9.00%)
S-8	Sugar, glucose, syrup. Total sugars: 78.3%
S-9	Saccharose, inverted sugar

**Table 2:** δ^13^C Isotopic range: examples of some C4 and C3 plant origin.

Metabolic Pathway	Plant Examples	δ13C	Method/Analysis Type
C4	Corn (maize), Sugar cane	−8‰ ÷ 13‰	AOAC 998.12, EA-IRMS
C3	Rice, Beet, Wheat, Chicory	−22‰ ÷ −30‰	Raezke 2008, LC-IRMS

**Table 3:** Effect of different candy boards and laboratories over the tested parameters.

		Acidity (meq/kg Candy)	Sucrose (g/100 g Candy)	Fructose (g/100 g Candy)	Glucose (g/100 g Candy)	5-HMF (mg/kg Candy)	pH	Water (g/100 g Candy)
Candy board (CB)	S-1	0.8 ± 0.1	70 ± 2	10 ± 1	10 ± 1	3.0 ± 0.5	6.2 ± 0.2	2 ± 1
	S-2	0.9 ± 0.1	74 ± 4	2.5 ± 0.3	4.3 ± 0.4	20 ± 11	4.8 ± 0.3	4 ± 1
	S-3	0.6 ± 0.1	78 ± 4	0.4 ± 0.3	4.4 ± 0.4	1.3 ± 0.4	5.5 ± 0.5	4 ± 1
	S-4	2.3 ± 0.2	75 ± 3	7.6 ± 0.3	7.8 ± 0.3	18 ± 8	4.4 ± 0.2	2 ± 1
	S-5	0.8 ± 0.1	77 ± 6	3.7 ± 0.3	2.0 ± 0.1	3.2 ± 0.5	4.8 ± 0.3	3 ± 1
	S-6	4.5 ± 0.3	nd	32 ± 1	40 ± 1	58 ± 5	3.9 ± 0.1	14 ± 1
	S-7	2.8 ± 0.3	76 ± 4	6 ± 1	5 ± 1	18 ± 6	4.1 ± 0.4	3 ± 1
	S-8	0.6 ± 0.1	70 ± 3	nd	2.4 ± 0.3	1 ± 1	5.6 ± 0.4	4 ± 1
	S-9	0.7 ± 0.1	75 ± 4	8 ± 1	5.3 ± 0.5	2.2 ± 0.5	6.3 ± 0.3	2 ± 1
*p*-Value ^A^ (*n* = 27)		<0.001	<0.001	<0.001	<0.001	<0.001	<0.001	<0.001
Laboratory (Lab)	Portugal	1.5 ± 1.2	67 ± 24	8 ± 8	9 ± 11	11 ± 17	5 ± 1	3 ± 4
	Slovakia	1.5 ± 1.3	67 ± 24	8 ± 9	9 ± 11	18 ± 21	5 ± 1	5 ± 4
	Slovenia	1.7 ± 1.4	65 ± 23	7 ± 9	9 ± 11	14 ± 16	5 ± 1	5 ± 3
*p*-value ^B^ (*n* = 81)		0.514	0.809	0.934	0.999	0.057	0.082	<0.001
CB × Lab *p*-value ^C^ (*n* = 243)		<0.001	<0.001	<0.001	<0.001	<0.001	<0.001	<0.001

^A^ *p*-Values lower than 0.001 indicate a significant difference in the corresponding parameter for at least one candy brand. ^B^ *p*-Values lower than 0.001 indicate a significant difference in the corresponding parameter for at least one laboratory. ^C^ *p*-Values lower than 0.001 indicate a significant interaction between the two factors (ST and FT); therefore, the statistical classification from the multiple comparison tests could not be presented; nd—not detected.

**Table 4:** Values of δ^13^C for candy board samples and their δ^13^C protein fractions by AOAC 998.12 (EA-IRMS), and the δ^13^C individual saccharides composition determined by LC-IRMS.

	Samples							
	S-1	S-2	S-3	S-4	S-5	S-6	S-7	S-9
δ^13^C_cb_ (‰)	−25.61	−26.36	−26.00	−25.23	−26.81	−11.82	−23.52	−23.61
δ^13^C_p_ (‰)	−25.63	−26.17	−26.18	−26.36	−26.81	−11.66	−22.51	−25.50
Δδ^13^C_cd-p_ (‰)	−0.02	0.19	−0.18	−0.12	0.00	0.15	1.01	−1.89
δ^13^C_diss_(‰)	−29.03	−25.40	−26.89	−28.92	-	−12.52	−25.40	−29.08
δ^13^C_triss_(‰)	-	−25.35	-	-	-	-	−25.41	-
Δδ^13^C_f-g_(‰)	0.18	−1.3	0.23	1.51	−31.2	−2.15	0.18	−5.59
Δδ^13^C_max_(‰)	2.68	−1.43	1.01	3.55	−31.2	−2.15	0.18	23.54
Diss (%)	62.3	14.3	4.50	74.5	-	3.30	13.5	74.7
Triss (%)	-	14.3	-	-	-	-	13.4	-
C4 (%)	0.20	0.00	1.10	0.80	0.00	100.0	0.00	25.3
C3 (%)	99.0	99.8	98.9	99.2	100	0.00	100.0	74.7

Abbreviations: δ^13^C_cb_—δ^13^C of candy board; δ^13^C_p_—δ^13^C of protein; δ^13^C_diss_—δ^13^C of disaccharides; δ^13^C_tri_—δ^13^C of trisaccharides; Δδ^13^C_cd-p_—difference between δ^13^C of candy board and δ^13^C of protein; Δδ^13^C_f-g_—difference between δ^13^C of fructose and δ^13^C of glucose. The analysis of the C3-C4 sugar origin was not performed for sample S8.

**Table 5:** Texture parameters of the candy boards analyzed.

Samples	Hardness/g
S-1	302.4 ± 44.5
S-2	74.2 ± 17.5
S-3	528.8 ± 7.1
S-4	474.4 ± 28.7
S-5	83.3 ± 21.3
S-6	35.6 ± 6.3
S-7	202.0 ± 20.1
S-8	230.3 ± 14.6
S-9	274.0 ± 17.2

## Data Availability

Data are contained within this article.
